# Digital screening for atrial fibrillation ready for prime time? Lessons learned from eBRAVE‐AF

**DOI:** 10.1002/ctm2.1151

**Published:** 2022-12-28

**Authors:** Michael Schreinlechner, Konstantinos D. Rizas, Axel Bauer

**Affiliations:** ^1^ University Hospital for Internal Medicine III Medical University of Innsbruck Innsbruck Austria; ^2^ Medizinische Klinik und Poliklinik I LMU University Hospital Munich Munich Germany

1

Atrial fibrillation (AF) is associated with significant morbidity and mortality that can be prevented by early detection and intervention.^1^ Digital screening strategies offer an effective way to detect unknown AF and have the potential to be widely applicable to the general population. Three large‐scale consumer‐oriented studies demonstrated the great potential of smart devices for the detection of unknown AF (Table 1).^2^, ^3^, ^4^ However, due to lack of randomisation or a control group, the added benefit over usual care remained unclear. In addition, participants were selected based on ownership of brand‐specific devices rather than risk criteria. As a result, participants were young and not representative of a population at risk. Although these studies recruited an impressive total of over one million participants, only 0.07% of all participants were ultimately diagnosed with AF.

**TABLE 1:** Real‐world studies using smart devices for atrial fibrillation (AF) detection

	Apple heart study^4^	Huawei heart study^3^	Fitbit heart study^2^	eBRAVE‐AF study^5^
Design	Observational	Observational	Observational	Randomised
Sponsor	Industry	Industry	Industry	Academic
Participants	Customers	Customers	Customers	Health insurance policyholders 50–90 years; CHA2DS2‐VASc score of ≥1 (men) or ≥2 (women)
Sample size (*n*)	419 297	187 912	455 699	5551
Age (median)	40 years	35 years	47 years	66 years
CHA2DS2‐VASc score	Not reported^a^	1 (mean)	1 (mean)	3 (median)
Screening type	Passive	Passive	Passive	Active
AF confirmation	7d ECG patch	MAFA hospital	7d ECG patch	14d ECG loop
Abnormal PPG evaluation by ECG	4.8:1 (21%)	1.6:1 (63%)	4.5:1 (22%)	1:1 (100%)
Primary endpoint	AF	AF	AF	AF treated with OAC
AF detection rate	0.04%	0.12%	0.08%	1.68% (digital arm) versus 0.89% (usual care)^b^

Abbreviations: eBRAVE‐AF, eHealth‐based Bavarian Alternative Detection of Atrial Fibrillation; ECG, electrocardiogram; OAC, oral anticoagulation; PPG, photoplethysmography.

^a^ 13% (55 277 of 419 297) CHA2DS2‐VASc score ≥2.

^b^ Given for first study phase. Detection rate of AF leading to OAC (primary endpoint) was 1.33% versus 0.63%.

With the eHealth‐based Bavarian Alternative Detection of Atrial Fibrillation (eBRAVE‐AF) study, we performed the first randomised, healthcare‐based trial to demonstrate the efficacy of a digital AF screening strategy in an elderly population at risk.^5^ The study was designed as a prospective, pragmatic, site‐less, digital, randomised trial with enrolment across Germany.^6^ In the trial, 5551 policyholders of a large German health insurance company (median age 65 years; 31% women; median CHA_2_DS_2_‐VASc 3) without known AF and without prescription of oral anticoagulation (OAC) were randomly assigned either to a digital screening strategy (*n* = 2860) or usual care (*n* = 2691). For digital screening, participants used a certified app (Preventicus Heartbeats, Preventicus GmbH, Jena, Germany) on their own smartphones to perform 1‐min photoplethysmographic (PPG) self‐measurements of their pulse waves. Abnormal PPG findings were verified by a 14‐day electrocardiogram patch. In contrast to previous studies, the primary endpoint required prescription of OAC due to newly diagnosed AF by an independent physician. Within 6 months of follow‐up, digital screening strategy could more than double the detection rate of treatment‐relevant AF compared to usual care with an odds ratio of 2.12 (95% confidence interval [CI] 1.19–3.76; *p* = .010). In the second study phase after crossover with reverse assignment to the screening strategies, digital screening was again associated with a 2.75‐fold (95% CI 1.42–5.34; *p* = .003) increased detection rate of treatment‐relevant AF. In conclusion, eBRAVE‐AF was the first trial that could demonstrate the efficacy of digital screening to detect treatment‐relevant AF directly compared to usual care.

Should digital AF screening now be generally recommended? We do not think so, as the answer to the most important question is still missing. eBRAVE‐AF was a diagnostic study that was neither intended to investigate the impact of digital screening on clinical outcomes nor adequately powered to do so. The key question of whether the implementation of a digital AF screening strategy can reduce the morbidity and mortality associated with AF is still unknown. The answer to this question will require a large‐scale randomised trial with a long follow‐up. Several issues must first be clarified.

First, the appropriate target population will need to be defined, as the success of any screening strategy depends critically on the selection of the right screening candidates. Therefore, effectiveness from both a patient and health care perspective will need to be taken into account. Underlying prevalence of the disease in the target population is a key factor, which in the case of AF is mainly influenced by age. Accordingly, the application of an age threshold, possibly in combination with other risk factors, seems mandatory.

Second, enrolment strategies will need to be developed in order to motivate people to participate. In eBRAVE‐AF, 5551 (8.2%) out of 67 488 invited policyholders followed the invitation to take part in the trial. Some may consider this number to be low, but it should be taken into account that enrolment was purely digital without personal patient contact. There is currently no benchmark for digital onboarding in such a randomised study. In any case, it will be important to understand the factors that influenced enrolment in order to refine recruitment strategies. Moreover, a possible selection bias in recruitment should be considered. Participants in eBRAVE‐AF were private health insurance policyholders whose socioeconomic status and education level might differ from those of the general population. Future strategies should aim to engage people from all socioeconomic and educational backgrounds.

Third, digital technologies should be easy to use for older people. In eBRAVE‐AF, increased age was not a negative factor, neither with regard to the quality or quantity of the PPG self‐measurements nor with regard to the effectiveness of the digital AF screening.

Fourth, the detection of AF should be closely linked to its treatment. The complexity of AF and its comorbidities requires a holistic approach to disease management that goes beyond the issue of OAC. AF detection may trigger a diagnostic work‐up for AF‐associated comorbidities, including individualised treatment approaches that may include management of heart failure, arterial hypertension or coronary artery disease.

It becomes clear that translating digital AF screening into evidence‐based clinical practice is a major challenge that requires a collaborative effort among all stakeholders, including physicians, public health providers, industry partners and patients. However, digital screening strategies may have a tremendous potential to improve patients’ outcomes.

## CONFLICT OF INTEREST

The authors declare they have no conflicts of interest.
